# Identification of A Novel Missense Mutation in The Norrie Disease Gene: The First Molecular Genetic Analysis and Prenatal Diagnosis of Norrie Disease in An Iranian Family

**DOI:** 10.22074/cellj.2018.5090.

**Published:** 2018-03-18

**Authors:** Farah Talebi, Farideh Ghanbari Mardasi, Javad Mohammadi Asl, Ali Lashgari, Freidoon Farhadi

**Affiliations:** 1 Ahvaz Welfare Organization, Ahvaz, Iran; 2 Department of Midwifery, Shoushtar Faculty of Medical Science, Shoushtar, Iran; 3 Department of Medical Genetics, Faculty of Medicine, Ahvaz Jundishapur University of Medical Science, Ahvaz, Iran; 4 Department of Ophthalmology, Faculty of Medicine, Shahid Beheshti University of Medical Science, Tehran, Iran; 5 Department of Social Science, Islamic Azad University of Shoushtar, Shoushtar, Iran

**Keywords:** Norrie Disease, NDP, Novel Mutation

## Abstract

Norrie disease (ND) is a rare X-linked recessive disorder, which is characterized by congenital blindness and, in several cases, accompanied with mental retardation and deafness. ND is caused by mutations in NDP, located on the proximal short arm of the X chromosome (Xp11.3). The disease has been observed in many ethnic groups worldwide, however, no such case has been reported from Iran. In this study, we present the molecular analysis of two patients with ND and the subsequent prenatal diagnosis (PND). Screening of NDP identified a hemizygous missense mutation (p.Ser133Cys) in the affected male siblings of the family. The mother was the carrier for the mutation (p.Ser133Cys). In a subsequent chorionic amniotic pregnancy, we carried out PND by sequencing NDP in the chorionic villi sample at 11 weeks of gestation. The fetus was carrying the mutation and thus unaffected. This is the first mutation report and PND of an Iranian family with ND, and highlights the importance of prenatal diagnostic screening of this congenital disorder and relevant genetic counseling.

## Introduction

Norrie disease (ND, MIM 310600) is an X-linked recessive disorder affecting male off spring by developing bilateral leucocorea (BL) in early infancy or even at birth. BL is due to primary retinal dysplasia and results in total retinal detachment and vitreous hemorrhage. At least 30-50% of cases reported have various degrees of intellectual disability (ID) with psychotic features and approximately 25% develop progressive sensorineural deafness, however, usually in late childhood (1). 

In addition to ND, the norrie disease protein gene (NDP) mutations have been reported in four other distinct retinopathies, namely persistent hyperplastic primary vitreous (PHPV), XL-familial exudative vitreous retinopathy (XL-FEVR), retinopathy of prematurity (ROP) and Coats’ disease, suggesting a common molecular pathogenicpathway. However, ID and hearing loss (HL) are not features of these allelic disorders (2).

ND is genetically homogeneous and caused by mutations in NDP, located on chromosome Xp11.3 (3) and encoding the 133-amino-acid long protein norrin (4). This protein has high homology to transforming growth factors with similar cysteine knot structure. Although the exact function of norrin is unknown, it has been suggested for it to play a crucial role in stria-vascularis, and vascular development of the retina and cerebellum (4, 5).

 Several studies screening *NDP* for causal variants have been reported in different ethnic groups from numerous countries (2), however, to the best of our knowledge, there are no reports regarding mutations in *NDP* in patients of Iranian origin. Herein, we report an Iranian ND family with a novel mutation in *NDP*, confirmed by ophthalmologic findings and subsequent prenatal genetic testing by chorionic villus sampling (CVS). 

###  Case report

**Fig.1:**
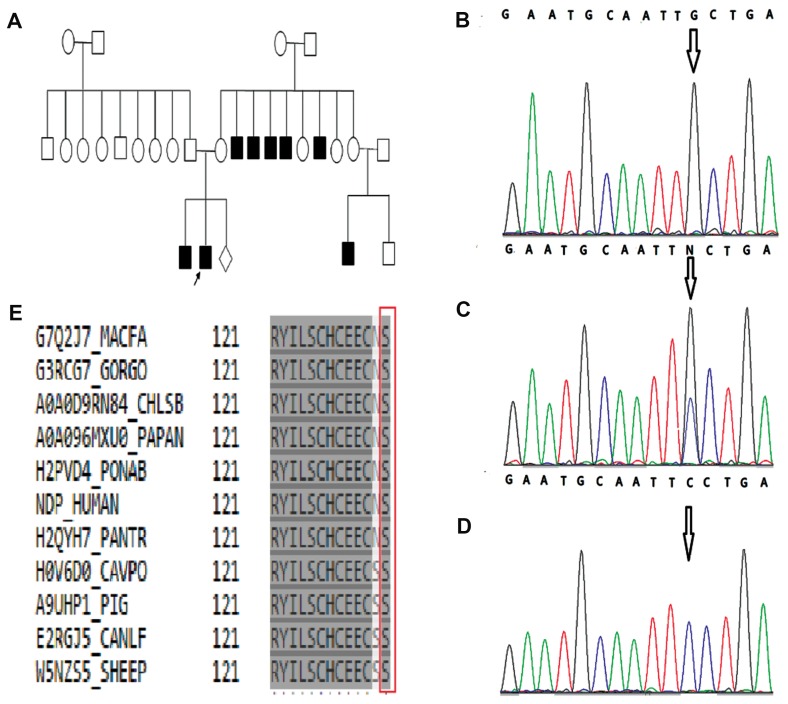
An overview of the genetic analysis of the Iranian ND family. A. Pedigree of the family with ND shows eight affected individuals. The patients are shown in black. Electropherogram analysis, B. Partial sequences of NDP in the patient shows cosegregation of the mutation with the phenotype, C. Electropherogram of the mother, D. Electropherogram of CVS from fetus. Position of variant is marked with arrow (black), and E. Conservation analysis. Protein alignment shows conservation of the amino acid sequence of NDP at position 133 among mammalian species (evolutionarily conserved residue shown in the vertical red box. ND; Norrie disease and NDP; Norrie disease protein.

A family (Fig .1A) with 27 members in three generations had a member (case III-11) with the classic symptoms of the disease. The proband was a 5-year-old Iranian male who had been blind in both eyes since infancy. Based on clinical evaluations, he had a normal hearing ability and normal psychomotor development. Pupils were not responsive to light and both retinas were invisible. Both parents of the patient were normal. The other members of the family had lived in Khuzestan province, located in South-West Iran. Although four of the affected individuals had normal intelligence (II-14, II-16, III-1, III-4), three (II-11, II-12, II-13) were mentally deficient. The mode of inheritance in the family was X-linked recessive. The other six affected members of this family were confirmed to have Norrie disease based on clinical and ophthalmic examinations. 

 We obtained peripheral blood samples with informed consent and analyzed DNA samples of the index patient, his sibling, his mother and 200 unrelated healthy controls for causal variants in coding exons of *NDP* using a method described in detail previously. Genomic DNA was extracted from peripheral leukocytes of the each participant, by using a standard salting out method and PCR amplification of the 2 coding exons (exons 2 and 3) of *NDP* was conducted by using oligonucleotide primer pairs (6). 

 Amplified DNA fragments of the two coding exons (exon 1 is 5´UTR) were sequenced by using dye terminator cycle sequencing andan automated sequencer (ABI 373A). Sequence analysis of the proband revealed a single nucleotide change at codon 133 (TCC to TGC), which causes a missense mutation (p.Ser133Cys). We did not analyze the other members of the family. 

 As illustrated in figure, sequencing of DNA showed that the proband and his brother were hemizygous for the mutation (Fig .1B), and the mother was a heterozygote carrier (Fig .1C). The mother was therefore counseled for the risk in her next pregnancy. A fetal ultrasound at 11 weeks revealed chorionic amniotic male fetus in her next pregnancy. Following counseling, a trans-abdominal CVS was conducted. Direct sequencing of polymerase chain reaction (PCR) products of fetal DNA from CVS demonstrated that the fetus was unaffected (Fig .1D). This mutation occurs in a conserved region of the protein (Fig .1E). Analysis of the 200 unrelated healthy controls revealed the absence of the mutation. 

## Discussion

The present ND family revealed a distinct genetic defect with a novel missense mutation in a manner expected for an X-linked recessive genetic disorder. This is not only the first study reporting an Iranian family with Norrie disease, but also the first to report a mutation at position 133 of norrin. Interestingly, this mutation occurs in a conserved region of the protein and responsible for disulphide bonds of the preserved cysteine knot motif of norrin, which may have a deleterious effect on its structure and function. Additionally, this change results in the substitution of a nonpolar amino acid to a polar one, thus possibly altering its isoelectric point and consequently affecting its function in physiological conditions. 

So far, over 140 different mutations of *NDP* have been described in the HGMD database (http://www.hgmd. cf.ac.uk/ac/all.php). A diversity of mutations has been detected in *NDP* in ND families, including nonsense and indel mutations resulting in truncated proteins, splicing defect mutations and initiation codon mutation. Clinical features in these genetically diagnosed patients are also variable, with patients showing severe ocular disease with variable hearing impairment and intellectual disability.

The mutation (p. Ser133Cys) identified in the proband with classical ND shows intra-familial variability in the appearance of extra-ocular symptoms, as the six uncles with congenital blindness had evidence of HL or ID. It is thus valuable to establish whether a relationship exists between the phenotype and the genotype, since the results obtained to this date show no such correlation. This inter and intra-familial phenotypic variability of *NDP* mutation carriers and lack of a strong genotype-phenotype correlation has led to many authors proposing the role of unknown genetic or epigenetic factors modulating the phenotypic appearance of ND (7-9). 

## Conclusion

This is the first genetic screening and prenatal diagnosis of Norrie disease in an Iranian family. This study highlights the importance of *NDP* screening for clinical diagnosis of ND and to identify inherited from sporadic cases, which is essential before prenatal diagnosis and genetic counseling can be offered to couples at risk.
